# Traumatische Kindheitserlebnisse und Herzgesundheit am Beispiel von Erwachsenen mit angeborenem Herzfehler

**DOI:** 10.1007/s00115-025-01898-4

**Published:** 2025-09-24

**Authors:** Britta Stapel, Friederieke Löffler, Mechthild Westhoff-Bleck, Ivo Heitland, Kai G. Kahl

**Affiliations:** 1 Klinik für Psychiatrie, Sozialpsychiatrie und Psychotherapie, Medizinische Hochschule Hannover, Carl-Neuberg Str. 1, 30625 Hannover, Deutschland; 2 Klinik für Kardiologie und Angiologie, Medizinische Hochschule Hannover, Hannover, Deutschland

**Keywords:** Epikardiales Fettgewebe, Depression, Körperliche Aktivität, Adverse Kindheitserlebnisse, Prävention, Epicardial adipose tissue, Depression, Physical activity, Adverse childhood experiences, Prevention

## Abstract

**Hintergrund:**

Adverse Kindheitserlebnisse (ACE) sind ein Risikofaktor für eine ungünstige Lebensweise, psychische Störungen und kardiometabolische Erkrankungen. Über welche Mechanismen diese Risiken mediiert werden, ist nicht abschließend untersucht.

**Fragestellung:**

Besteht ein Zusammenhang zwischen ACE und Herzgesundheit bei Erwachsenen mit angeborenem Herzfehler (EMAH)?

**Methoden:**

Insgesamt 609 EMAH wurden eingeschlossen. Soziodemografische Parameter, ACE (Childhood Trauma Questionnaire [CTQ]), depressive Symptomatik (Hospital Anxiety and Depression Scale [HADS]) und sportliche Aktivität wurden erfragt. Patienten erhielten eine vollständige kardiologische Untersuchung einschließlich der Messung des epikardialen Fettgewebes (EAT) mittels Echokardiographie. Es wurden Bootstrapping-Mediationsanalysen durchgeführt, mit ACE als Prädiktor, depressiven Symptomen und körperlicher Aktivität als Mediatoren und EAT als abhängige Variable.

**Ergebnisse:**

Der CTQ-Gesamtwert zeigte einen signifikanten indirekten Effekt auf EAT, der seriell durch depressive Symptomatik und körperliche Aktivität mediiert wurde (CTQ → HADS-D → Sport → EAT; a*d*b2 = 0,0171, 95 %-[Konfidenzintervall] KI [0,0080, 0,0285]). Vergleichbare signifikante Effekte zeigten sich für die einzelnen CTQ-Domänen (emotionale/körperliche Vernachlässigung, emotionaler/körperlicher Missbrauch, sexuelle Traumatisierung).

**Diskussion:**

Wir zeigen, dass ACE mit verstärkten depressiven Symptomen einhergeht, die zu geringerer körperlicher Aktivität und vergrößertem EAT führen – einem Risikomarker für kardiale Ereignisse. Unsere Ergebnisse weisen auf einen zentralen Mechanismus hin, über den ACE die kardiovaskuläre Gesundheit beeinträchtigt, und zeigen mehrere Ansatzpunkte für primär- und sekundärpräventive Interventionen in einer multimodalen Therapie von EMAH.

**Zusatzmaterial online:**

Zusätzliche ergänzende Tabellen zu Stichproben und deren Ergebnisse sind in der Online-Version dieses Artikels (10.1007/s00115-025-01898-4) enthalten.

## Abstract (en)

**Background:**

Adverse childhood experiences (ACE) are a risk factor for an unfavorable lifestyle, mental disorders and cardiometabolic diseases. However, the mechanisms through which these risks are mediated have not been conclusively investigated.

**Research question:**

Is there a connection between ACE and cardiovascular health in adults with congenital heart disease (ACHD)?

**Methods:**

A total of 609 ACHD were included. Sociodemographic parameters, ACE (Childhood Trauma Questionnaire, CTQ), depressive symptoms (Hospital Anxiety and Depression Scale, HADS) and physical activity were assessed. Patients underwent a full cardiological examination including the measurement of epicardial adipose tissue (EAT) using echocardiography. Bootstrapped mediation analyses were performed with ACE as the predictor, depressive symptoms and physical activity as mediators and EAT as the dependent variable.

**Results:**

The CTQ sum score showed a significant indirect effect on EAT, which was serially mediated by depressive symptoms and physical activity (CTQ → HADS-D → Exercise → EAT; a*d*b2 = 0.0171, 95% confidence interval, CI 0.0080, 0.0285). Comparable significant effects were found for the individual CTQ domains (emotional/physical neglect, emotional/physical abuse, sexual trauma).

**Discussion:**

We show that ACE are associated with increased depressive symptoms, which in turn lead to reduced physical activity and increased EAT, a risk marker for cardiac events. Our findings point to a key mechanism through which ACE impair cardiovascular health and highlight several targets for primary and secondary preventive interventions within a multimodal treatment approach for ACHD.

Angeborene Herzfehler sind die häufigsten Geburtsdefekte. Heute erreichen über 98 % der Betroffenen das Erwachsenenalter. Somit bilden Erwachsene mit angeborenem Herzfehler (EMAH) eine wachsende Patientenpopulation, gekennzeichnet zum einen durch ein erhöhtes Risiko für kardiovaskuläre Folgeerkrankungen, zum anderen aber auch durch psychosoziale Probleme. EMAH berichten vermehrt von adversen Kindheitserlebnissen („adverse childhood experiences“, ACE) und psychischen Erkrankungen. Diese Studie untersucht mittels serieller Mediationsanalysen den Zusammenhang zwischen ACE, Depression, körperlicher Aktivität und strukturellen Herzveränderungen bei EMAH.

## Hintergrund

Angeborene Herzfehler sind mit einer Jahresprävalenz von 0,9–1 % aller Lebendgeburten die häufigsten angeborenen Defekte [1]. Sie umfassen ein Spektrum anatomischer Fehlbildungen, das von spezifischen Läsionen wie dem Ventrikelseptumdefekt bis zu komplexen Läsionen wie der Transposition großer Arterien reicht [2]. Dank frühzeitiger Diagnose und Fortschritten in der Herzchirurgie und interventioneller Kardiologie hat sich die Überlebensrate von Patienten mit angeborenem Herzfehler deutlich verbessert. Die Gruppe der Erwachsenen mit angeborenem Herzfehler (EMAH) wird daher stetig größer und wird auf ca. 1,2 Mio. Patienten in Europa geschätzt [3].

Psychosoziale Probleme, z. B. herzbezogene Ängste, Bedenken hinsichtlich Sterblichkeit und Kinderwunsch, Anpassung an medizinische Prozeduren, Adhärenz und Beschäftigungsfähigkeit, treten ebenfalls stärker in den Vordergrund [4].

Erwachsene mit angeborenem Herzfehler sind eine Risikopopulation für traumatische Kindheitserlebnisse („adverse childhood experiences“, ACE). In einer aktuellen Studie wurde eine erhöhte Rate an emotionaler Vernachlässigung und emotionalem Missbrauch gefunden und selbstberichteter Kindesmissbrauch im Childhood Trauma Questionnaire (CTQ) war nicht nur mit einer verminderten Lebensqualität, sondern auch mit einer verminderten Herzfunktion assoziiert [5].

Über welchen Weg ACE das Risiko für kardiovaskuläre Erkrankungen im Erwachsenenalter erhöht, ist bislang nicht abschließend untersucht. In einer Voruntersuchung mit 201 EMAH konnten wir zeigen, dass ACE einen indirekten Einfluss auf strukturelle Herzveränderungen nahm. Mithilfe von Mediationsanalysen konnten wir darstellen, dass ACE mit einer höheren Rate depressiver Symptome, niedrigerer körperlicher Aktivität und einer vermehrten Akkumulation epikardialen Fettgewebes (EAT) assoziiert war [6].

Epikardiales Fettgewebe ist ein ektopisches Fettgewebe in unmittelbarer Nähe des Herzmuskels und der Koronargefäße, das parakrine Wirkungen auf umliegende Gewebe ausübt [7].

Epikardiales Fettgewebe korreliert mit Arteriosklerose unabhängig vom Vorliegen anderer Risikofaktoren, mit myokardialer Ischämie, kardiovaskulären Ereignissen, Hochrisikoplaques und Messgrößen für die linksventrikuläre Struktur und Funktion, z. B. dem linksventrikulären (LV) enddiastolischen Volumen, verschlechterter diastolischer Füllung und einer LV diastolischen Dysfunktion [8, 9]. Die LV-Dysfunktion spielt eine wichtige Rolle in der Entwicklung einer Herzinsuffizienz, die als Ursache für die Sterblichkeit bei EMAH zunehmend erkannt wird [10].

Die vorliegende Studie wurde an einer größeren Patientenpopulation mit 609 EMAH durchgeführt. Die Hypothesen waren:

- Der CTQ-Gesamtscore ist mit Depressivität, körperlicher Aktivität und strukturellen Herzveränderungen assoziiert (Replikation der Ergebnisse an einem größeren Studienkollektiv).
- Die einzelnen Domänen des CTQ (körperliche/emotionale Vernachlässigung, körperlicher/emotionaler Missbrauch, sexuelle Traumatisierung) sind ebenfalls – mediiert über Depression und körperliche Aktivität – mit strukturellen Herzveränderungen assoziiert.
- Der CTQ-Gesamtscore und die CTQ-Domänenwerte sind mit der funktionellen Leistungsfähigkeit, gemessen als kardiopulmonale Belastungsfähigkeit, assoziiert.

## Methoden

### Studiendesign und Stichprobenbeschreibung

Die hier ausgewerteten Daten stammen aus dem PsyConHeart-Projekt, einer seit 2013 fortlaufendenden Registerstudie, die die Rolle psychiatrischer Symptome und Störungen auf den Verlauf der kardialen Erkrankung von EMAH untersucht [4, 11–14]. Die Studie wurde vom zuständigen Ethikkomitee bewilligt und gemäß der Deklaration von Helsinki durchgeführt. Alle Patienten wurden über die Studie aufgeklärt und unterzeichneten eine Einverständniserklärung. Der Einschluss von Patienten erfolgte über die EMAH-Ambulanz der Medizinischen Hochschule Hannover. Einschlusskriterien waren wie folgt: Diagnose eines kongenitalen Herzfehlers, ausreichende Deutschkenntnisse, um die eingesetzten Fragebögen zu beantworten, und ein Alter von ≥ 18 Jahren. Ausschlusskriterien waren Schwangerschaft und Instabilität der kardialen Erkrankung.

### Kardiologische Untersuchungen

Die kardiologischen Untersuchungen wurden von einer kardiologischen Fachärztin mit Spezialisierung auf die Behandlung von EMAH durchgeführt.

Die EAT-Schichtdicke, welche einen etablierten Prädiktor für kardiometabolisches Risiko darstellt, wurde echokardiographisch bestimmt [15]. Die Messungen erfolgten mittels zweidimensionaler enddiastolischer Ansicht in der parasternalen langen und kurzen Achse. Die Bestimmung der EAT-Schichtdicke erfolgte an der freien rechten Ventrikelwand senkrecht zum Aortenannulus.

Die Bestimmung der maximalen Sauerstoffaufnahme (VO_2_ max) erfolgte mittels einer symptomlimitierten kardiopulmonalen Belastungstestung, die in aufrechter Körperposition auf einem Fahrradergometer durchgeführt wurde, wobei die Belastung kontinuierlich alle 2 min um 25 W gesteigert wurde. VO_2_ max dient als Maß für die maximale funktionelle Leistungsfähigkeit und stellt eine etablierte, objektive Kenngröße der maximalen funktionellen Kapazität dar.

### Psychometrische Untersuchungen

Depression- und Angstsymptome wurde mittels der Hospital Anxiety and Depression Scale (HADS; [16]) ermittelt. Vorherige Studien haben gezeigt, dass ein an EMAH-Patienten angepasster Cut-off-Wert von > 5 auf der Depressionsunterskala des HADS das Vorliegen einer moderaten oder schweren Depression zuverlässig prädestiniert [17]. ACE wurde retrospektiv mittels des Childhood Trauma Questionnaire (CTQ) gemessen, der fünf Domänen abbildet (emotionaler Missbrauch, körperlicher Missbrauch, sexueller Missbrauch, emotionale Vernachlässigung und körperliche Vernachlässigung) [18, 19]. Die körperliche Aktivität wurde mittels modifizierter Cuppett Lattin Scale [20] erfragt und der aktuelle Raucherstatus wurde dokumentiert. Bei allen eingesetzten Skalen handelt es sich um Selbstauskunftsinstrumente.

### Stichprobe

Inkludiert wurden alle Patienten, von denen vollständige Daten für die in die jeweiligen Mediationsanalysen aufgenommenen Variablen vorlagen. Daraus ergab sich eine Stichprobengröße von *N* = 609 (Gesamtstichprobe) für die Analysen mit EAT als abhängige Variable und eine Stichprobengröße von *N* = 310 (Teilstichprobe) für Analysen mit der abhängigen Variable VO_2_ max. Die Charakterisierung beider Stichproben bezüglich demographischer, kardialer und psychometrischer Faktoren ist in der ergänzenden Tabelle S1 online zusammengefasst.

Da es sich beim PsyConHeart-Projekt um eine fortlaufende Registerstudie handelt, beinhaltet die Gesamtstichprobe Daten von Patienten, die bereits in einer vorrangegangenen Publikation eingeschlossen waren (*N* = 195), in der der Effekt von Kindheitstraumatisierung auf EAT analysiert wurde [6]. Entsprechende Mediationsanalysen, die diese Patienten exkludieren, sind in den ergänzenden Materialien online dargestellt (ergänzende Resultate, ergänzende Tabelle S4 für eine Charakterisierung der Stichproben und ergänzende Tabelle S5 für die Ergebnisse der entsprechenden Mediationsanalysen mit EAT als abhängige Variable). Die entsprechende Stichprobe inkludierte *N* = 414 Patienten.

### Statistik

**Abb. 1:**
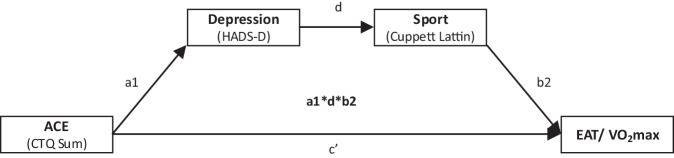
A‑priori vorgeschlagenes Mediationsmodell zur Verbindung traumatischer Kindheitserlebnisse („adverse childhood experiences“ [*ACE*], Prädiktor, unabhängige Variable) mit kardialen Parametern (epikardiales Fettgewebe [*EAT*] oder maximale Sauerstoffaufnahme [*VO*_*2*_ *max*], abhängige Variable) über die seriellen Mediatoren Depression und körperliche Bewegung (*Sport*). *CTQ Sum* Childhood Trauma Questionnaire Gesamtscore, *Cuppett Lattin* Cuppett Lattin Scale,* HADS-D *Hospital Anxiety and Depression Scale, deutsche Version

Alle statistischen Analysen wurden mit SPSS Statistics Version 29.0 (IBM Corp., Armonk, NY, USA) durchgeführt. Zur Durchführung der Mediationsanalysen wurde PROCESS V5.0 verwendet [21, 22], um die unstandardisierten Pfadkoeffizienten der totalen, direkten und indirekten Effekte zu ermitteln. Hierbei wurde Modell 6, das zwei serielle Mediatoren einschließt, verwendet (Abb. 1). In allen Mediationsanalysen wurden Alter, Geschlecht und Body-Mass-Index (BMI) als Kovariate eingeschlossen. Bootstrapping mit 10.000 Iterationen mit heteroskedastizitätskonsistenten Standardfehlern (SE) wurde durchgeführt, um 95 %-Konfidenzintervalle (95 %-KI) und Inferenzstatistiken zu berechnen. Effekte wurden als signifikant erachtet, wenn das KI Null nicht einschloss. Für alle statistischen Tests wurde ein α‑Niveau von 0,05 angenommen.

## Resultate

### Mediationsanalysen zum Effekt von ACE auf EAT

**Abb. 2:**
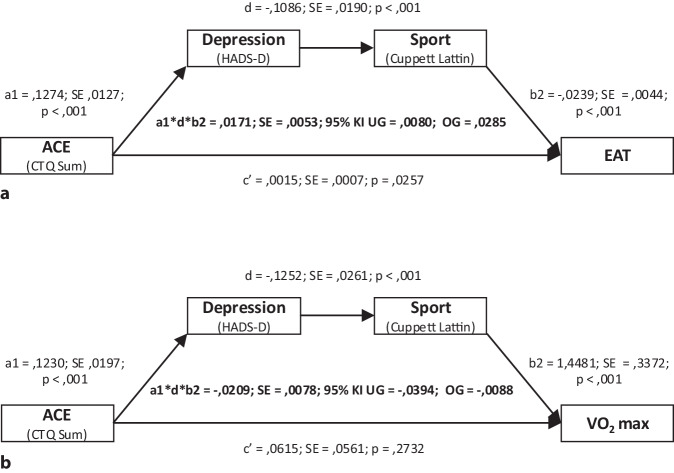
Ergebnisse der Prüfung der jeweiligen seriellen Mediationsmodelle (Modell 6, korrespondierend zu Abb. 1). Der Effekt traumatischer Kindheitserlebnisse („adverse childhood experiences“, *ACE*) über die seriellen Mediatoren Depression und körperliche Bewegung (*Sport*) auf die Dicke des epikardialen Fettgewebes (*EAT*, **a**) und die maximale Sauerstoffaufnahme (*VO*_*2*_* max*, **b**) ist dargestellt. Unstandandardisierte Regressionskoeffizienten sowie entsprechende Standardfehler (*SE*) und *p*-Werte für die entsprechenden Pfade sind gezeigt. Die Statistik zu den jeweiligen indirekten Effekten findet sich im Zentrum der Diagramme und umfasst vollständig standardisierte Regressionskoeffizienten, SEs sowie die zugehörigen unteren (*UG*) und oberen (*OG*) Grenzen des 95 %-Konfidenzintervalls (*KI*). *CTQ Sum* Childhood Trauma Questionnaire Gesamtscore, *Cuppett Lattin* Cuppett Lattin Scale,* HADS-D *Hospital Anxiety and Depression Scale, deutsche Version

Es wurden serielle Mediationsanalysen durchgeführt, um zu überprüfen, ob ACE (CTQ-Gesamtscore) die Akkumulation von EAT vorhersagt und ob der direkte Pfad durch die seriellen Mediatoren Depression (HADS-D) und körperliche Bewegung (Cuppett Lattin Scale) mediiert wird. Die unstandardisierten Pfadkoeffizienten der totalen, direkten und indirekten Effekte sind in der ergänzenden Tabelle S2 online und in Abb. 2a dargestellt.

Es zeigte sich, dass ACE einen signifikanten indirekten Effekt auf EAT, mediiert über Depressionswert und körperliche Bewegung, hat (a1*d*b2 = 0,0171; SE = 0,0053; 95 %-KI: UG = 0,0080; OG = 0,0285).

Ähnliche Ergebnisse zeigten sich in den entsprechenden seriellen Mediationsanalysen für die jeweiligen Domänen des CTQ. Für alle Domänen fanden sich signifikante indirekte Effekte auf EAT, mediiert durch Depression und körperliche Bewegung (emotionaler Missbrauch: a1*d*b2 = 0,0154; SE = 0,0049; 95 %-KI: UG = 0,0072; OG = 0,0267; körperlicher Missbrauch: a1*d*b2 = 0,0088; SE = 0,0035; 95 %-KI: UG = 0,0030; OG = 0,0167; sexueller Missbrauch: a1*d*b2 = 0,0093; SE = 0,0038; 95 %-KI: UG = 0,0034; OG = 0,0179; emotionale Vernachlässigung: a1*d*b2 = 0,0166; SE = 0,0049; 95 %-KI: UG = 0,0081; OG = 0,0274; körperliche Vernachlässigung: a1*d*b2 = 0,0091; SE = 0,0033; 95 %-KI: UG = 0,0038; OG = 0,0167; ergänzende Tabelle S2 online).

### Mediationsanalysen zum Effekt von ACE auf VO_2_ max

Ähnlich wie für EAT zeigte sich hinsichtlich der auf das Körpergewicht normalisierten VO_2_ max ein signifikanter indirekter Effekt von ACE mediiert über Depressionswert und körperliche Bewegung (a1*d*b2 = −0,209; SE = 0,0078; 95 %-KI: UG = −0,0394; OG = −0,0088; ergänzende Tabelle S3 online und Abb. 2b).

Darüber hinaus fanden sich für die jeweiligen Domänen des CTQ ähnliche signifikante, durch Depression und körperliche Aktivität vermittelte, indirekte Effekte auf die VO_2_ max (emotionaler Missbrauch: a1*d*b2 = −0,208; SE = 0,0084; 95 %-KI: UG = −0,0398; OG = −0,0080; körperlicher Missbrauch: a1*d*b2 = −0,0105; SE = 0,0064; 95 %-KI: UG = −0,0030; OG = −0,0004; sexueller Missbrauch: a1*d*b2 = −0,0147; SE = 0,0073; 95 %-KI: UG = −0,0320; OG = −0,0034; emotionale Vernachlässigung: a1*d*b2 = −0,0189; SE = 0,0069; 95 %-KI: UG = −0,0348; OG = −0,0081; körperliche Vernachlässigung: a1*d*b2 = −0,0095; SE = 0,0051; 95 %-KI: UG = −0,0212; OG = −0,0014; ergänzende Tabelle S3 online).

## Diskussion

Unsere Studie mit 609 EMAH repliziert die Ergebnisse der Vorstudie und zeigt, dass ACE mit einer Zunahme von EAT im Erwachsenenalter (strukturelle Herzveränderung) verbunden ist. Wichtige Mediatoren in diesem Prozess sind die Entwicklung depressiver Symptome und behaviorale Änderungen mit verminderter körperlicher Aktivität.

Dabei sagte nicht nur der Gesamtscore des CTQ im seriellen Mediationsmodell die Zunahme von EAT voraus. Auch jede einzelne CTQ-Domäne war mit einer Zunahme von EAT, mediiert über depressive Symptome und körperliche Inaktivität, assoziiert.

Darüber hinaus konnten wir an einer Unterstichprobe den Zusammenhang von ACE und objektiv gemessener funktioneller Leistungsfähigkeit zeigen.

### Neurobiologische Hypothesen zur Assoziation von ACE mit EAT

Frühkindlicher Stress, z. B. in Form von ACE, hat tiefgreifende und dauerhafte Auswirkungen auf die Entwicklung des Gehirns und trägt zu langfristigen neurologischen und verhaltensbezogenen Veränderungen bei. Befunde auf neurobiologischer Ebene zeigten eine Volumenminderung des Hippokampus, die mit Beeinträchtigungen des Gedächtnisses und der Emotionskontrolle in Verbindung steht. ACE führt zu einer Sensibilisierung der Amygdala mit der Folge ausgeprägterer Angstreaktionen. Darüber hinaus kann ACE die Entwicklung des präfrontalen Kortex stören, was Entscheidungsfindung, Planung und Impulskontrolle beeinträchtigt [23]. In einer Studie von Congio et al. wurde gezeigt, dass ACE mit verringerter körperlicher Aktivität, kognitiven Einschränkungen und erhöhten Entzündungswerten einhergeht [24].

Traumatische Kindheitserlebnisse können die Aktivität von Neurotransmittersystemen beeinflussen, was mitverursachend für die erhöhte Rate an Angststörungen und Depression sein kann. Darüber hinaus erhöht ACE die Stressanfälligkeit und macht die Betroffenen anfälliger für eine überschießende Stressreaktion mit konsekutivem Hyperkortisolismus [23].

Epikardiales Fettgewebe ist ein kortisolresponsibles Fettgewebe, das unter einer erhöhten Kortisolexkretion an Größe zunimmt. In einer Studie zu den Auswirkungen der Depression auf ektopes Fettgewebe wurde gezeigt, dass das Volumen an epikardialem und intraabdominalem Fettgewebe mit der Nebennierengröße korreliert. Diese ist ein Proxy-Parameter für die Kortisolbelastung über die Lebensspanne. Diese Ergebnisse legen nahe, dass die bei Depression häufig anzutreffende Dysregulation der Hypothalamus-Hypophysen-Nebennierenachse mit der Vergrößerung des EAT in Verbindung steht [25].

### Ansätze für Primär- und Sekundärprävention

Die Ergebnisse unserer Studie reihen sich in eine zunehmende Forschungsliteratur zu den gesundheitlichen Konsequenzen traumatischer Kindheitserlebnisse ein. In einer Metaanalyse, die 19 Studien mit > 20 Mio. Teilnehmenden einschloss, zeigten Grummitt et al., dass ACE ein führender Risikofaktor für die Entwicklung psychischer und körperlicher Erkrankungen ist. Die Autoren kommen zu dem Schluss, dass ACE ein hochrelevanter, veränderlicher Faktor ist, der allein in den USA mit über 439.000 Todesfällen, einer erhöhten Rate sexuell übertragbarer Erkrankungen, illegalem Drogenkonsum, Rauchen und Bewegungsmangel assoziiert ist [26].

In unserer Studie wurde eine Risikopopulation für kindliche Traumatisierung, insbesondere für emotionale Vernachlässigung und Missbrauch, betrachtet [6]. Zu der erhöhten ACE-Rate bei EMAH können verschiedene Prozesse beitragen, z. B. Stigmatisierung des Kindes durch die chronische Erkrankung, mögliche Einschränkungen in der Beschulung oder Belastung der Eltern durch häufige Arztbesuche und Klinikaufenthalte [6]. Unsere Ergebnisse legen daher nahe, schon bei Diagnosestellung des angeborenen Herzfehlers im Kindesalter die Eltern in die Therapieplanung und auch in mögliche Hilfen für die Alltagsbewältigung zu integrieren.

#### Primärpräventive Interventionen

Die Betrachtung von ACE als vermeidbaren Risikofaktor, der zu einer schlechteren Prognose der kardiologischen Grunderkrankung und früher Sterblichkeit beiträgt, könnte dazu anregen, Interventionen und Finanzmittel auf die primäre und sekundäre Prävention ungünstiger kindlicher Aufwuchsbedingungen zu lenken. Die Verhinderung der Exposition gegenüber ACE gelingt am ehesten durch politische Interventionen, die strukturelle Lösungen für die Einschränkungen bieten, denen Familien mit z. B. Kindern mit angeborenem Herzfehler ausgesetzt sind. Eine gegebenenfalls spezielle und erschwingliche Kinderbetreuung und/oder Elternurlaub bei medizinischen Maßnahmen sind geeignet, um den elterlichen Stress zu verringern und Eltern besser in die Lage zu versetzen, ihre Kinder angemessen und einfühlsam zu betreuen.

Auch Hausbesuchsdienste, die Familien über die Erkrankung und die möglichen Einschränkungen ihres Kindes aufklären, sie unterstützen und verfügbare Informationen über sozialmedizinische Hilfen zur Verfügung stellen, sind denkbar. So konnte in anderen Kontexten gezeigt werden, dass Hausbesuchsdienste Kindesmissbrauch, Vernachlässigung und Besuche in der Notaufnahme verringern [27]. In den USA, wo solche Hausbesuchsdienste eine gewisse Verbreitung gefunden haben, erreichen diese Dienste allerdings nur 3–6 % der Familien, die davon profitieren könnten [27].

Evidenzbasierte Programme wie das Programm „Safe Environment for Every Kid“ – eine kurze, skalierbare Intervention im Rahmen der pädiatrischen Grundversorgung – sind vielversprechend bei der Prävention von Kindesmisshandlung. Das Programm hat gezeigt, dass eine Schulung pädiatrischer Grundversorgungsärzte zur Erkennung und Behandlung von Risikofaktoren für Kindesmisshandlung in Familien die Raten von Kindesmisshandlung im Vergleich zur Standardversorgung reduziert [28].

#### Sekundärpräventive Interventionen

Sekundärpräventive Ansätze könnten mit einem standardisierten Screening für psychopathologische Auffälligkeiten bereits im Kindes- und Jugendalter bei Risikokindern beginnen. Im Erwachsenenalter könnte ein Screening für Angsterkrankungen und Depression, z. B. in Form des 2 × 2-Fragen-Tests (GAD‑2 kombiniert mit PHQ-2), ein Minimalstandard sein. Dieses Vorgehen wird mittlerweile bei einigen kardiologischen Risikopatienten empfohlen [29].

Neben der Diagnose und Therapie manifester psychischer Erkrankungen sollte bei Risikopatienten eine Lebensstilberatung durchgeführt werden, die die drei Domänen „gesunde Ernährung“, „Suchtmittel“ und „sportliche Aktivität“ beinhaltet. Bezüglich sportlicher Interventionen konnten wir in einer kontrollierten Studie bei Patienten mit Depression zeigen, dass ein strukturiertes, monitoriertes und personalisiertes Trainingsprogramm nicht nur das Therapieergebnis einer leitliniengerechten Depressionsbehandlung bessert, sondern auch EAT verringert [30].

## Fazit für die Praxis

- Adverse Kindheitserlebnisse (ACE) stellen einen Risikofaktor für die Entwicklung struktureller Herzveränderungen im Erwachsenenalter dar.
- Prognostische und veränderbare Mediatoren sind die Entwicklung einer Depression und ein inaktiver Lebensstil mit geringer körperlicher Fitness.
- Durch geeignete Screeningmethoden können diese Faktoren im Erwachsenenalter diagnostiziert und spezifisch interveniert werden.
- Primärpräventiv können politische Entscheidungsträger strukturelle Erleichterungen für Familien mit Kindern mit angeborenem Herzfehler schaffen.
- Die hier gewonnen Ergebnisse könnten auch auf andere chronische Erkrankungen mit Begin in der Kindheit übertragen werden. Hierzu bedarf es weiterer Forschung, die auch Aspekte der Transition berücksichtigt.

## Supplementary Information

Tabelle S1 Charakterisierung von Gesamtstichprobe und Teilstichprobe; Tabelle S2 Traumatische Kindheitserlebnisse sind indirekt, über die seriellen Mediatoren Depression und körperliche Bewegung, mit einer verstärkten Akkumulation von epikardialem Herzfettgewebe assoziiert. Tabelle S3 Traumatische Kindheitserlebnisse sind indirekt, über die seriellen Mediatoren Depression und körperliche Bewegung, mit verminderter maximaler Sauerstoffaufnahme assoziiert. Tabelle S4 Charakterisierung der Stichprobe; Tabelle S5 Retrospektiv berichtete Kindheitstraumatisierung ist indirekt, über die seriellen Mediatoren Depression und körperliche Bewegung, mit einer verstärkten Akkumulation von epikardialem Herzfettgewebe assoziiert.

## Data Availability

Die der Studie zugrunde liegenden Daten sind aus Gründen der Sensibilität nicht öffentlich zugänglich. Sie können jedoch auf begründete Anfrage bei der korrespondierenden Autorin angefordert werden.
