# Supplementary material for: Traumatic childhood experiences and cardiovascular health using the example of adults with congenital heart disease
Source: Nervenarzt. 2025 Sep 24;97(1):44–9. [Article in German] doi: 10.1007/s00115-025-01898-4 (PMC12808155; doi:10.1007/s00115-025-01898-4)
Supplement: Supplementary file 1 — Tabelle S1 Charakterisierung von Gesamtstichprobe und Teilstichprobe; Tabelle S2 Traumatische Kindheitserlebnisse sind indirekt, über die seriellen Mediatoren Depression und körperliche Bewegung, mit einer verstärkten Akkumulation von epikardialem Herzfettgewebe assoziiert. Tabelle S3 Traumatische Kindheitserlebnisse sind indirekt, über die seriellen Mediatoren Depression und körperliche Bewegung, mit verminderter maximaler Sauerstoffaufnahme assoziiert. Tabelle S4 Charakterisierung der Stichprobe; Tabelle S5 Retrospektiv berichtete Kindheitstraumatisierung ist indirekt, über die seriellen Mediatoren Depression und körperliche Bewegung, mit einer verstärkten Akkumulation von epikardialem Herzfettgewebe assoziiert. [file 115_2025_1898_MOESM1_ESM.pdf]

**Ergänzende Tabellen zu Artikel: Traumatische Kindheitserlebnisse und Herzgesundheit am Beispiel von Erwachsenen mit angeborenem Herzfehler**

**Ergänzende Tabelle S1: Charakterisierung von Gesamtstichprobe und Teilstichprobe**

| **Variable** | **Gesamtstichprobe (N = 609)** | **Teilstichprobe (N = 310)** |
|---|---|---|
| Alter in Jahren (MW ± SE) | 36,5 ± 11,8 | 35,5 ± 11,2 |
| Geschlecht (N, %) | | |
| weiblich | 291 (47,8%) | 149 (48,1%) |
| männlich | 318 (52,2%) | 161 (51,9%) |
| BMI in kg/m$^2$ (MW ± SE) | 25,7 ± 5,0 | 25,1 ± 4,7 |
| Körperliche Bewegung (MW ± SE) | 3,52 ± 1,52 | 3,57 ± 1,505 |
| Rauchen (N, %) | 93 (15,3%) | 54 (17,4%) |
| NYHA-Klasse (N, %) | | |
| NYHA-I | 400 (65,7%) | 223 (71,9%) |
| NYHA-II | 113 (18,6%) | 57 (18,4%) |
| NYHA-II | 34 (5,6%) | 15 (4,8%) |
| NYHA-IV | 2 (0,3%) | 0 (0,0%) |
| Bethesda-Klasse (N, %) | | |
| Bethesda I | 114 (18,7%) | 69 (22,3%) |
| Bethesda II | 209 (34,3%) | 112 (36,1%) |
| Bethesda III | 283 (46,5%) | 128 (41,3%) |
| EAT in cm (MW ± SE) | 0,3945 ± 0,1940 | 0,3937 ± 0,2081 |
| $VO_2$ max in ml/(min*kg) (MW ± SE) | 24,56 ± 9,79 | 24,56 ± 9,79 |
| LVEF in % (MW ± SE) | 56,7 ± 9,0 | 57,0 ± 8,9 |
| NTproBNP in ng/l (MW ± SE) | 243,3 ± 352,6 | 211,5 ± 285,2 |
| Kardiovaskuläre Medikation (N, %) | | |
| ACE- oder AT-Blocker (Sartane) | 218 (36%) | 112 (36,2%) |
| Aldosteron-Antagonist | 82 (13,5%) | 43 (13,9%) |
| Beta-Blocker | 203 (33,3%) | 101 (32,6%) |
| Calciumantagonist | 33 (5,4%) | 12 (3,9%) |
| Diuretikum | 69 (11,4%) | 26 (8,4%) |
| Psychopharmakon (N, %) | 38 (6,3%) | |
| CTQ Summenwert (MW ± SE) | 32,9 ± 10,0 | 32,4 ± 9,2 |
| CTQ EM Schweregrad (N, %) | | |
| kein-minimal | 501 (82,3%) | 259 (83,5%) |
| minimal-moderat | 63 (10,3%) | 33 (10,6%) |
| moderat-schwer | 19 (3,1%) | 7 (2,3%) |
| schwer-extrem | 26 (4,3%) | 11 (3,5%) |
| CTQ KM Schweregrad (N, %) | | |
| kein-minimal | 571 (93,8%) | 259 (94,2%) |
| minimal-moderat | 15 (2,5%) | 8 (2,6%) |
| moderat-schwer | 11 (1,8%) | 5 (1,6%) |
| schwer-extrem | 12 (2,0%) | 5 (1,6%) |
| CTQ SM Schweregrad (N, %) | | |
| kein-minimal | 559 (91,8%) | 285 (91,9%) |
| minimal-moderat | 19 (3,1%) | 12 (3,9%) |
| moderat-schwer | 18 (3,0%) | 7 (2,3%) |
| schwer-extrem | 13 (2,1%) | 6 (1,9%) |
| CTQ EV Schweregrad (N, %) | | |
| kein-minimal | 420 (69,0%) | 219 (70,6%) |
| minimal-moderat | 132 (21,7%) | 65 (21,0%) |
| moderat-schwer | 39 (6,4%) | 17 (5,5%) |
| schwer-extrem | 18 (3,0%) | 9 (2,9%) |

**Fortsetzung Ergänzende Tabelle S1: Charakterisierung von Gesamtstichprobe und Teilstichprobe**

| Variable | Gesamtstichprobe (N = 609) | Teilstichprobe (N = 310) |
|---|---|---|
| CTQ KV Schweregrad (N, %) | | |
| kein-minimal | 466 (76,5%) | 239 (77,1%) |
| minimal-moderat | 87 (14,3%) | 49 (15,8%) |
| moderat-schwer | 37 (6,1%) | 17 (5,5%) |
| schwer-extrem | 19 (3,1%) | 5 (1,6%) |
| HADS-D Summenwert (MW ± SE) | 3,65 ± 3,36 | 3,38 ± 3,35 |
| HADS-D Schweregrad (N, %)* | | |
| normal/leicht (≤ 5} | 462 (75,9%) | 244 (78,7%) |
| moderate/schwer {> 5) | 147 (24,8%) | 66 (21,3%) |

Die Tabelle zeigt Mittelwerte (MW) mit Standardabweichung (SE) oder N-Zahlen und Prozente.
ACE, *Angiotensin Converting Enzyme*; AT, Angiotensin-Rezeptor; BMI, Body-Mass-Index; CTQ, Kindheitstrauma Fragebogen; EAT, epikardiales Fettgewebe; EM, emotionaler Missbrauch, EV, emotionale Vernachlässigung; HADS-D, Depressionscore mittels *Hospital Anxiety and Depression Scale*; KM, körperlicher Missbrauch; KV, körperliche Vernachlässigung; LVEF, linksventrikuläre Auswurffraktion; NYHA, *New York Heart Association* Klassifizierung; $VO_2$ max, maximale Sauerstoffaufnahme normalisiert auf Körpergewicht. *Cut-off Werte definiert nach [1]

**Ergänzende Tabelle S2: Traumatische Kindheitserlebnisse sind indirekt, über die seriellen Mediatoren Depression und körperliche Bewegung, mit einer verstärkten Akkumulation von epikardialem Herzfettgewebe assoziiert.**

| Unabhängige Variable | Abhängige Variable | Statistik | | | | |
|---|---|---|---|---|---|---|
| | | Pfad | RK | SE | 95% KI | p-Wert |
| CTQ Sum (N = 609) | EAT | a1 | 0,1274 | 0,0127 | UG = 0,1024; OG = 0,1524 | < 0.001 |
| | | d | -0,1086 | 0,0190 | UG = -0,1459; OG = -0,0713 | < 0,001 |
| | | b2 | -0,0239 | 0,0044 | UG = -0,0325; OG = -0,0154 | < 0,001 |
| | | c‘ | 0,0015 | 0,0007 | UG = 0,0002; OG = 0,0029 | = 0,026 |
| CTQ EM (N = 609) | EAT | a1 | 0,3499 | 0,0395 | UG = 0,2723; OG = 0,4275 | < 0,001 |
| | | d | -0,1092 | 0,0187 | UG = -0,1459; OG = -0,0724 | < 0,001 |
| | | b2 | -0,0240 | 0,0044 | UG = -0,0325; OG = -0,0154 | < 0,001 |
| | | c‘ | 0,0047 | 0,0021 | UG = 0,0007; OG = 0,0088 | = 0,022 |
| CTQ KM (N = 609) | EAT | a1 | 0,3626 | 0,0763 | UG = 0,2129; OG = 0,05124 | < 0.001 |
| | | d | -0,1110 | 0,0179 | UG = -0,1462; OG = -0,0758 | < 0.001 |
| | | b2 | -0,0241 | 0,0044 | UG = -0,0327: OG = -0,0155 | < 0.001 |
| | | c‘ | 0,0051 | 0,0037 | UG = -0,0021; OG = 0,0123 | = 0,166 |
| CTQ SM (N = 609) | EAT | a1 | 0,3575 | 0,0676 | UG = 0,2247; OG = 0,4902 | < 0.001 |
| | | d | -0,1068 | 0,0180 | UG = -0,1422; OG = -0,0715 | < 0.001 |
| | | b2 | -0,0238 | 0,0044 | UG = -0,0324; OG = -0,0153 | < 0.001 |
| | | c‘ | 0,0038 | 0,0033 | UG = -0,0026; OG = 0,0102 | = 0,248 |
| CTQ EV (N = 609) | EAT | a1 | 0,3218 | 0,0328 | UG = 0,2573; OG = 0,3863 | < 0.001 |
| | | d | -0,1086 | 0,0189 | UG = -0,1458; OG = -0,0714 | < 0.001 |
| | | b2 | -0,0239 | 0,0044 | UG = -0,0325; OG = -0,0154 | < 0.001 |
| | | c‘ | 0,0033 | 0,0018 | UG = -0,0002; OG = 0,0067 | = 0,063 |
| CTQ KV (N = 609) | EAT | a1 | 0,2966 | 0,0583 | UG = 0,1822; OG = 0,4110 | < 0.001 |
| | | d | -0,1090 | 0,0180 | UG = -0,1443; OG = -0,0737 | < 0.001 |
| | | b2 | -0,0240 | 0,0044 | UG = -0,0325; OG = -0,0154 | < 0.001 |
| | | c‘ | 0,0028 | 0,0028 | UG = -0,0027; OG = 0,0083 | = 0,323 |

Dargestellt sind die Ergebnisse der seriellen Mediationsmodelle (Modell 6, siehe Abb. 1). Neben den dargestellten Prädiktoren und EAT als abhängige Variable wurden Depressionsscore (HADS-D Summenwert) und körperliche Bewegung (Cuppett Lattin Scale) als serielle Mediatoren getestet.
CTQ, Kindheitstrauma Fragebogen; EAT, epikardiales Fettgewebe; EM, emotionaler Missbrauch; EV, emotionale Vernachlässigung; KI, Konfidenzintervall; KM, körperlicher Missbrauch, KV, körperliche Vernachlässigung; OG, obere Grenze; RK, Regressionskoeffizient; SE: Standardabweichung; SM, sexueller Missbrauch; Sum, Summenwert, UG, untere Grenze.

**Ergänzende Tabelle S3: Traumatische Kindheitserlebnisse sind indirekt, über die seriellen Mediatoren Depression und körperliche Bewegung, mit verminderter maximaler Sauerstoffaufnahme assoziiert.**

| Unabhängige Variable | Abhängige Variable | Statistik | | | | |
|---|---|---|---|---|---|---|
| | | Pfad | RK | SE | 95% KI | p-Wert |
| CTQ Sum (N = 310) | $VO_2$ max | a1 | 0,1230 | 0,0197 | UG = 0,0843; OG = 0,1617 | < 0,001 |
| | | d | -0,1252 | 0,0261 | UG = -0,1766; OG = -0,0738 | < 0,001 |
| | | b2 | 1,4481 | 0,3372 | UG = 0,7846; OG = 2,1115 | < 0,001 |
| | | c‘ | 0,0615 | 0,0561 | UG = -0,0488; OG = 0,1719 | = 0,273 |
| CTQ EM (N = 310) | $VO_2$ max | a1 | 0,3723 | 0,0587 | UG = 0,2568; OG = 0,4878 | < 0,001 |
| | | d | -0,1222 | 0,0261 | UG = -0,1736; OG = -0,0707 | < 0,001 |
| | | b2 | 1,4505 | 0,3377 | UG = 0,7859; OG = 2,1150 | < 0,001 |
| | | c‘ | 0,1272 | 0,1680 | UG = -0,2034; OG = 0,4578 | = 0,450 |
| CTQ KM (N = 310) | $VO_2$ max | a1 | 0,3187 | 0,1116 | UG = 0,0991; OG = 0,05383 | = 0,046 |
| | | d | -0,1317 | 0,0249 | UG = -0,1807; OG = -0,0827 | < 0,001 |
| | | b2 | 1,4414 | 0,3381 | UG = 0,7760: OG = 2,1067 | < 0,001 |
| | | c‘ | -0,0212 | 0,2901 | UG = -0,5922; OG = 0,5497 | = 0,942 |
| CTQ SM (N = 310) | $VO_2$ max | a1 | 0,5273 | 0,1302 | UG = 0,2711; OG = 0,7836 | = 0,001 |
| | | d | -0,1315 | 0,0252 | UG = -0,1812; OG = -0,0819 | < 0,001 |
| | | b2 | 1,4333 | 0,3377 | UG = 0,7688; OG = 2,0978 | < 0,001 |
| | | c‘ | 0,2270 | 0,3471 | UG = -0,4559; OG = 0,9100 | = 0,514 |
| CTQ EV (N = 310) | $VO_2$ max | a1 | 0,2809 | 0,0499 | UG = 0,1827; OG = 0,3791 | < 0,001 |
| | | d | -0,1248 | 0,0258 | UG = -0,1757; OG = -0,0740 | < 0,001 |
| | | b2 | 1,4511 | 0,3370 | UG = 0,7879; OG = 2,1142 | < 0,001 |
| | | c‘ | 0,1729 | 0,1390 | UG = -0,1007; OG = 0,4465 | = 0,215 |
| CTQ KV (N = 310) | $VO_2$ max | a1 | 0,2446 | 0,0901 | UG = 0,0673; OG = 0,4220 | = 0,007 |
| | | d | -0,1252 | 0,0249 | UG = -0,1742; OG = -0,0763 | < 0,001 |
| | | b2 | 1,4571 | 0,3375 | UG = 0,7929; OG = 2,1213 | < 0,001 |
| | | c‘ | 0,2446 | 0,2334 | UG = -0,2146; OG = 0,7039 | = 0,295 |

Dargestellt sind die Ergebnisse der seriellen Mediationsmodelle (Modell 6, siehe Abb. 1). Neben den dargestellten Prädiktoren und EAT als abhängige Variable wurden Depressionsscore (HADS-D Summenwert) und körperliche Bewegung (Cuppett Lattin Scale) als serielle Mediatoren getestet.

CTQ, Kindheitstrauma Fragebogen; EAT, epikardiales Fettgewebe; EM, emotionaler Missbrauch; EV, emotionale Vernachlässigung; KI, Konfidenzintervall; KM, körperlicher Missbrauch, KV, körperliche Vernachlässigung; OG, obere Grenze; RK, Regressionskoeffizient; SE: Standardabweichung; SM, sexueller Missbrauch; Sum, Summenwert; UG, untere Grenze; $VO_2$ max, maximale Sauerstoffaufnahme normalisiert auf Körpergewicht.

**Ergänzende Ergebnisse**

Die folgenden Tabellen zeigen Ergebnisse basierend auf einer Teilstichprobe, in der Patienten, die in der vorrausgegangenen Publikation [2] bereits eingeschlossen waren, exkludiert wurden. Die resultierende Stichprobe umfasst Daten von N = 414 Patienten und bestätigt die Ergebnisse, die im Hauptteil der Publikation dargestellt sind. Die Charakterisierung der Stichprobe ist in der ergänzenden Tabelle S4 zusammengefasst und die Ergebnisse der entsprechenden Mediationsanalysen finden sich in der ergänzenden Tabellen S5 (abhängige Variable EAT).

**Ergänzende Tabelle S4: Charakterisierung der Stichprobe**

| **Variable** | **Stichprobe (N = 414)** |
|---|---|
| Alter in Jahren (MW ± SE) | 36,5 ± 11,8 |
| Geschlecht (N, %) | |
| weiblich | 205 (49,5%) |
| männlich | 209 (50,5%) |
| BMI in kg/m$^2$ (MW ± SE) | 24,8 ± 4,9 |
| Körperliche Bewegung (MW ± SE) | 3,56 ± 1,50 |
| Rauchen (N, %) | 42 (10,1%) |
| NYHA-Klassifikation (N, %) | |
| NYHA-I | 267 (64,5%) |
| NYHA-II | 72 (17,4%) |
| NYHA-II | 23 (5,6%) |
| NYHA-IV | 2 (0,5%) |
| Bethesda-Klassifikation (N, %) | |
| Bethesda I | 46 (11,1%) |
| Bethesda II | 146 (35,3%) |
| Bethesda III | 219 (52,9,5%) |
| EAT in cm (MW ± SE) | 0,3751 ± 0,1854 |
| $VO_2$ max in ml/(min*kg) (MW ± SE) | 23,67 ± 10,26 |
| LVEF in % (MW ± SE) | 57,3 ± 8,2 |
| NTproBNP in ng/l (MW ± SE) | 234,9 ± 337,5 |
| Kardiovaskuläre Medikation (N, %) | |
| ACE- oder AT-Blocker (Sartane) | 137 (33,2%) |
| Aldosteron-Antagonist | 58 (14,1%) |
| Beta-Blocker | 135 (32,8%) |
| Calciumantagonist | 21 (5,1%) |
| Diuretikum | 41 (10%) |
| Psychopharmakon (N, %) | 16 (3,9%) |
| CTQ Summenwert (MW ± SE) | 32,3 ± 9,4 |
| CTQ EM Schweregrad (N, %) | |
| kein-minimal | 343 (82,9%) |
| minimal-moderat | 41 (9,9%) |
| moderat-schwer | 14 (3,4%) |
| schwer-extrem | 16 (3,9%) |
| CTQ KM Schweregrad (N, %) | |
| kein-minimal | 394 (95,2%) |
| minimal-moderat | 8 (1,9%) |
| moderat-schwer | 7 (1,7%) |
| schwer-extrem | 5 (1,2%) |
| CTQ SM Schweregrad (N, %) | |
| kein-minimal | 382 (92,3%) |
| minimal-moderat | 14 (3,4%) |
| moderat-schwer | 12 (2,9%) |
| schwer-extrem | 6 (1,4%) |

**Fortsetzung Ergänzende Tabelle S4: Charakterisierung der Stichprobe**

| **Variable** | **Stichprobe (N = 414)** |
|---|---|
| CTQ EV Schweregrad (N, %) | |
| kein-minimal | 288 (69,6%) |
| minimal-moderat | 86 (20,8%) |
| moderat-schwer | 28 (6,8%) |
| schwer-extrem | 12 (2,9%) |
| CTQ KV Schweregrad (N, %) | |
| kein-minimal | 322 (77,8%) |
| minimal-moderat | 52 (12,6%) |
| moderat-schwer | 27 (6,5%) |
| schwer-extrem | 13 (3,1%) |
| HADS-D Summenwert (MW ± SE) | 3,65 ± 3,36 |
| HADS-D Schweregrad (N, %)* | |
| normal/leicht (≤ 5} | 320 (77,3%) |
| moderate/schwer {> 5) | 94 (22,7%) |

Die Tabelle zeigt Mittelwerte (MW) mit Standardabweichung (SE) oder N-Zahlen und Prozente.

ACE, *Angiotensin Converting Enzyme*; AT, Angiotensin-Rezeptor; BMI, Body-Mass-Index; CTQ, Kindheitstrauma Fragebogen; EAT, epikardiales Fettgewebe; EM, emotionaler Missbrauch, EV, emotionale Vernachlässigung; HADS-D, Depressionscore mittels *Hospital Anxiety and Depression Scale*; KM, körperlicher Missbrauch; KV, körperliche Vernachlässigung; LVEF, linksventrikuläre Auswurffraktion; NYHA, *New York Heart Association* Klassifizierung; $VO_2$ max, maximale Sauerstoffaufnahme. *Cut-off Werte definiert nach [1]

**Ergänzende Tabelle S5: Retrospektiv berichtete Kindheitstraumatisierung ist indirekt, über die seriellen Mediatoren Depression und körperliche Bewegung, mit einer verstärkten Akkumulation von epikardialem Herzfettgewebe assoziiert.**

| Unabhängige Variable | Abhängige Variable | Statistik | | | | |
|---|---|---|---|---|---|---|
| | | **Pfad** | **RK** | **SE** | **95% KI** | **p-Wert** |
| CTQ Sum (N = 414) | EAT | a1 | 0,1336 | 0,0156 | UG = 0,1030; OG = 0,1643 | < 0.001 |
| | | d | -0,1152 | 0,0236 | UG = -0,1617; OG = -0,0687 | < 0,001 |
| | | b2 | -0,0244 | 0,0048 | UG = -0,0338; OG = -0,0151 | < 0,001 |
| | | c‘ | 0,0016 | 0,0008 | UG = 0,0001; OG = 0,0031 | = 0,043 |
| | | *a1*d*b2* | *0,0191* | *0,0058* | *UG = 0,0093; OG = 0,0323* | |
| CTQ EM (N = 414) | EAT | a1 | 0,3613 | 0,0464 | UG = 0,2701; OG = 0,4226 | < 0,001 |
| | | d | -0,1205 | 0,0233 | UG = -0,1664; OG = -0,0746 | < 0,001 |
| | | b2 | -0,0247 | 0,0047 | UG = -0,0341; OG = -0,0154 | < 0,001 |
| | | c‘ | 0,0060 | 0,0022 | UG = 0,0016; OG = 0,0104 | = 0,008 |
| | | *a1*d*b2* | *0,0187* | *0,0057* | *UG = 0,0091; OG = 0,0313* | |
| CTQ KM (N = 414) | EAT | a1 | 0,4305 | 0,1181 | UG = 0,1983; OG = 0,6627 | = 0,003 |
| | | d | -0,1163 | 0,0221 | UG = -0,1597; OG = -0,0728 | < 0,001 |
| | | b2 | -0,0247 | 0,0048 | UG = -0,0341: OG = -0,0153 | < 0,001 |
| | | c‘ | -0,0019 | 0,0052 | UG = -0,0121; OG = 0,0083 | = 0,710 |
| | | *a1*d*b2* | *0,0088* | *0,0043* | *UG = 0,0017; OG = 0,0186* | |
| CTQ SM (N = 414) | EAT | a1 | 0,2170 | 0,0888 | UG = 0,0423; OG = 0,3916 | = 0,015 |
| | | d | -0,1188 | 0,0219 | UG = -0,1619; OG = -0,0757 | < 0,001 |
| | | b2 | -0,0245 | 0,0048 | UG = -0,0339; OG = -0,0152 | < 0,001 |
| | | c‘ | 0,0069 | 0,0038 | UG = -0,0006; OG = 0,0144 | = 0,071 |
| | | *a1*d*b2* | *0,0061* | *0,0032* | *UG = 0,0009; OG = 0,0136* | |
| CTQ EV (N = 414) | EAT | a1 | 0,3229 | 0,0379 | UG = 0,2484; OG = 0,3975 | < 0,001 |
| | | d | -0,1146 | 0,0236 | UG = -0,1610; OG = -0,0682 | < 0,001 |
| | | b2 | -0,0245 | 0,0048 | UG = -0,0339; OG = -0,0151 | < 0,001 |
| | | c‘ | 0,0028 | 0,0019 | UG = -0,0010; OG = 0,0065 | = 0,148 |
| | | *a1*d*b2* | *0,0190* | *0,0055* | *UG = 0,0096; OG = 0,0308* | |
| CTQ KV (N = 414) | EAT | a1 | 0,3511 | 0,0674 | UG = 0,2185; OG = 0,4836 | < 0,001 |
| | | d | -0,1173 | 0,0225 | UG = -0,1615; OG = -0,0732 | < 0,001 |
| | | b2 | -0,0246 | 0,0048 | UG = -0,0340; OG = -0,0152 | < 0,001 |
| | | c‘ | 0,0024 | 0,0031 | UG = -0,0036; OG = 0,0084 | = 0,438 |
| | | *a1*d*b2* | *0,0125* | *0,0044* | *UG = 0,0052; OG = 0,0224* | |

Dargestellt sind die Ergebnisse der seriellen Mediationsmodelle (Modell 6, siehe Abb. 1). Neben den dargestellten Prädiktoren und EAT als abhängige Variable wurden Depressionsscore (HADS-D Summenwert) und körperliche Bewegung (Cuppett Lattin Scale) als serielle Mediatoren getestet. CTQ, Kindheitstrauma Fragebogen; EAT, epikardiales Fettgewebe; EM, emotionaler Missbrauch; EV, emotionale Vernachlässigung; KI, Konfidenzintervall; KM, körperlicher Missbrauch, KV, körperliche Vernachlässigung; OG, obere Grenze; RK, Regressionskoeffizient SE: Standardabweichung; SM, sexueller Missbrauch; Sum, Summenwert, UG, untere Grenze.

## Referenzen


1. Westhoff-Bleck M, Winter L, Aguirre Davila L, et al. Diagnostic evaluation of the hospital depression scale (HADS) and the Beck depression inventory II (BDI-II) in adults with congenital heart disease using a structured clinical interview: Impact of depression severity. Eur J Prev Cardiol. 2020;27(4):381-90.
2. Bertele S, Heitland I, Fraccarollo D, et al. Behavioral pathway to a broken heart: The link between adverse childhood experiences, depression, physical exercise and cardiovascular health. Front Psychiatry. 2022;13:1002143.
